# Multimodal Audiovestibular Assessment After Intratympanic Gentamicin for Vestibular Drop Attacks in Ménière’s Disease: A Case Report and Focused Review

**DOI:** 10.3390/diagnostics16121860

**Published:** 2026-06-16

**Authors:** Gabriela Cornelia Musat, Codrut Sarafoleanu, Mihai Alexandru Preda, Andreea Alexandra Mihaela Musat, Ionut Tanase

**Affiliations:** 1 Department of Otorhinolaryngology, Faculty of Dentistry, University of Medicine and Pharmacy “Carol Davila”, 050474 Bucharest, Romania; gabriela.musat@umfcd.ro (G.C.M.); codrut.sarafoleanu@umfcd.ro (C.S.); ionut.tanase@umfcd.ro (I.T.); 2 Doctoral School, University of Medicine and Pharmacy “Carol Davila”, 050474 Bucharest, Romania

**Keywords:** Ménière’s disease, Tumarkin crises, vestibular drop attacks, intratympanic gentamicin, caloric testing, video head impulse test, videonystagmography, vestibular deficit, audiovestibular evaluation

## Abstract

**Background and Clinical Significance:** Vestibular drop attacks are a rare but disabling manifestation of Ménière’s disease. This study aimed to demonstrate the value of multimodal audiovestibular assessment in documenting vestibular deficit after intratympanic gentamicin therapy. **Case presentation:** A 57-year-old woman with refractory Tumarkin crises underwent serial audiological and vestibular testing before and after intratympanic gentamicin treatment. Post-treatment testing demonstrated right-sided vestibular hypofunction with caloric areflexia, reduced vestibulo-ocular reflex gain, corrective saccades on video head impulse testing, and absent cervical vestibular-evoked myogenic potentials. Hearing thresholds showed transient deterioration with near-baseline recovery during follow-up. Vestibular drop attacks resolved completely. **Conclusions:** Multimodal audiovestibular assessment provides objective documentation of treatment-induced vestibular hypofunction after intratympanic gentamicin therapy in refractory Ménière’s disease.

## 1. Introduction

Ménière’s disease was first described in 1861 by the French physician Prosper Ménière [1]. Although its pathophysiology remains incompletely understood, the condition is associated with endolymphatic hydrops, characterized by an abnormal accumulation of endolymph in the membranous labyrinth. For this reason, Ménière’s disease (MD) is also referred to as idiopathic endolymphatic hydrops. Clinically, the disease is defined by recurrent episodes of vertigo, fluctuating sensorineural hearing loss, and aural fullness. These symptoms can severely impact quality of life and impair patients’ daily activities.

Among the most dramatic manifestations of MD are vestibular drop attacks (VDAs). These events are characterized by a sudden loss of postural control of vestibular origin, causing the patient to fall abruptly, without a prodrome or loss of consciousness. Although VDAs are not common in Ménière’s, when present, they represent one of the most dramatic and disabling manifestations. First described by Tumarkin in 1936, these attacks were subsequently named Tumarkin crises [2,3]. They are also referred to as otolithic crises or an otolithic catastrophe. The prevailing hypothesis is that these drop attacks result from sudden deformation of the otolithic organs (utricle and saccule), leading to inappropriate activation of the vestibulospinal reflexes [4]. The most important complications of VDAs are traumatic injuries. The unpredictability of Tumarkin drop attacks can compromise patient safety, leading to falls and physical trauma and profoundly affecting quality of life. In severe cases, these attacks can be a primary indication for ablative therapy.

This paper reports the case of a woman with MD complicated by Tumarkin drop attacks. Initial intratympanic corticosteroid treatment did not improve her symptoms. Later, intratympanic aminoglycoside therapy was given, leading to symptom remission. A multimodal vestibular assessment was used to evaluate the impact of gentamicin on her vestibular system.

Despite its efficacy, intratympanic gentamicin remains underutilized in some patients because clinicians may be concerned about cochlear toxicity and uncertain about the expected audiovestibular outcomes after treatment. Detailed post-treatment functional monitoring data remain relatively limited in the literature.

### Materials and Methods

Pure-tone audiometry was performed using standard air- and bone-conduction threshold measurements in a sound-treated booth. Videonystagmography (VNG), bithermal caloric testing, video head impulse testing (vHIT), and cervical vestibular-evoked myogenic potentials (cVEMPs) were performed using Interacoustics equipment (Interacoustics A/S, Middelfart, Denmark).

Bithermal caloric irrigation was performed using warm (44 °C) and cold (30 °C) stimulation, and the canal paresis was calculated according to the Jongkees formula. Video head impulse testing was used to evaluate the vestibulo-ocular reflex (VOR) gain and corrective saccades of the semicircular canals. Cervical vestibular-evoked myogenic potentials (cVEMPs) were recorded from the sternocleidomastoid muscles using air-conducted acoustic stimuli.

Audiovestibular assessments were performed before treatment and during follow-up after treatment.

## 2. Case Report

We present the case of a 57-year-old woman with no relevant past medical history who was examined in our clinic for a history of recurrent vertigo over about one year. Each episode lasted approximately one hour, occurred about once a month, and was associated with fluctuating hearing loss in the right ear, vertigo, tinnitus, nausea, and vomiting during the attacks.

**Figure 1:**
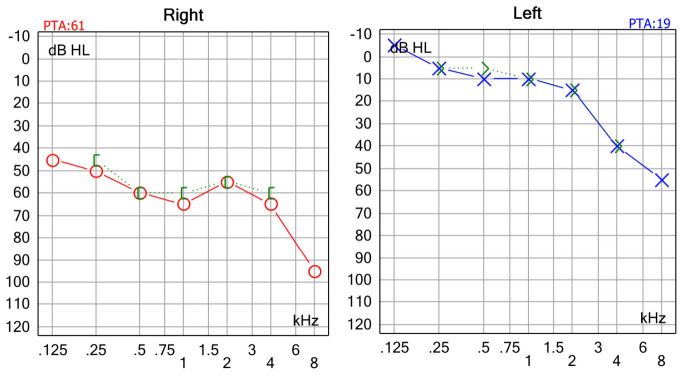
Pure-tone audiogram obtained at presentation. Red circles represent right-ear air-conduction thresholds, and blue crosses represent left-ear air-conduction thresholds. The dashed green line represents bone-conduction thresholds. Frequencies are displayed in kHz on the horizontal axis and hearing thresholds in dB HL on the vertical axis. The audiogram at presentation shows severe sensorineural hearing loss in the right ear and mild sensorineural hearing loss in the left ear.

On physical examination, we did not find any significant changes. The otorhinolaryngological (ENT) and neurological examinations were within normal limits. Clinical vestibular examination revealed no spontaneous nystagmus, a normal Romberg test, and no other abnormal vestibular signs at the time of examination. Pure-tone audiometry was performed using standard air- and bone-conduction testing, demonstrating severe sensorineural hearing loss in the right ear with a flat configuration and mild sensorineural hearing loss in the left ear with a sloping configuration (see Figure 1). Pure-tone average (PTA) was 19 for the left ear and 61 for the right ear.

The diagnosis of definite unilateral Ménière’s disease was established according to the 2015 Bárány Society/American Academy of Otolaryngology–Head and Neck Surgery (AAO-HNS) consensus criteria. The patient fulfilled the diagnostic criteria for definite unilateral Ménière’s disease, including recurrent spontaneous episodes of vertigo lasting between 20 min and 12 h; documented unilateral low- to mid-frequency sensorineural hearing loss; fluctuating aural symptoms, including tinnitus and aural fullness; and the absence of a better alternative vestibular diagnosis [5,6].

### 2.1. First-Line Therapy

Initial management consisted of medical therapy with acetazolamide (250 mg twice daily) and betahistine (24 mg twice daily) for a period of 6 months. Because symptoms persisted with only minimal improvement, intratympanic corticosteroid therapy was initiated.

The procedure was performed under local anesthesia. Topical anesthesia of the tympanic membrane was achieved by applying lidocaine to the external auditory canal. Under microscopic visualization, 0.5 mL of dexamethasone (4 mg/mL) was slowly injected into the middle ear cavity through the posteroinferior quadrant of the tympanic membrane using a 25-gauge spinal needle. After the injection, the patient remained supine with the treated ear facing upward for approximately 30 min and was instructed to avoid swallowing to facilitate diffusion of the medication through the round-window membrane. The procedure was well tolerated, and no immediate complications were observed.

After completion of intratympanic corticosteroid therapy, medical treatment with betahistine was continued. Complete remission of vertigo episodes was achieved and persisted for approximately one year.

### 2.2. Recurrence and Drop Attacks

After one year, the patient returned with a new symptom profile. She experienced sudden falls from standing height without warning, prodromal symptoms, or loss of consciousness. She denied true rotational vertigo and instead presented with abrupt collapses without any premonitory signs or symptoms. The new symptoms were considered consistent with the diagnosis of vestibular drop attacks (VDAs)/Tumarkin crises. Alternative causes of sudden falls were carefully considered. The patient denied loss of consciousness, palpitations, presyncopal symptoms, tonic–clonic movements, tongue biting, postictal confusion, or focal neurological deficits. Neurological examination was unremarkable, and the clinical presentation was not suggestive of syncope, epileptic seizures, transient ischemic attacks, orthostatic hypotension, or other central neurological disorders. The attacks were characterized by sudden postural collapse without impaired consciousness, consistent with vestibular drop attacks (Tumarkin crises). The episodes occurred every few days for approximately 3 weeks before the hospital presentation. The patient presented with multiple contusions all over the body.

A second course of intratympanic dexamethasone (0.5 mL of 4 mg/mL dexamethasone) was administered 7 days apart. Intratympanic corticosteroid therapy protocols vary considerably among institutions, with most regimens consisting of repeated transtympanic dexamethasone injections administered weekly over 3–4 weeks. In our clinic, we use a four-injection protocol. After the first injection, she did not experience any attack, which was considered a promising sign. Following the second injection, the patient experienced a severe drop attack leading to cranial trauma with a large extracranial hematoma.

### 2.3. Impact on Quality of Life

At this stage, the patient, otherwise a mentally balanced individual, developed marked anxiety. She was unable to continue her professional activities, required assistance with ambulation, and always wore a protective helmet. The impact on quality of life was substantial.

Given the severity and frequency of her VDAs, a decision was made to start vestibular ablation therapy with intratympanic gentamicin.

### 2.4. Definitive Therapy with Gentamicin

**Figure 2:**
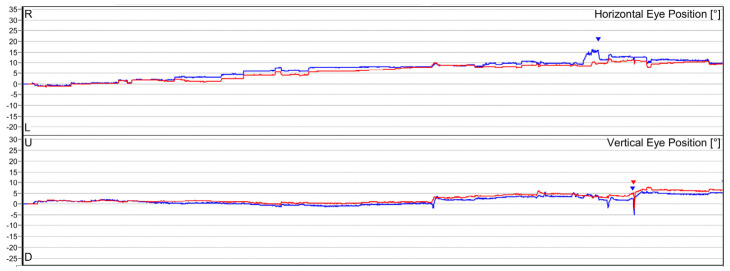
Video nystagmography (VNG) recording obtained at presentation, demonstrating the absence of spontaneous nystagmus. The upper panel shows horizontal eye position recordings, while the lower panel shows vertical eye position recordings (degrees) over time (seconds). Red and blue tracings represent recordings from each eye. No significant spontaneous horizontal or vertical ocular drift was observed during baseline examination. Eye movements were recorded using an Interacoustics videonystagmography system (Interacoustics A/S, Denmark).

**Figure 3:**
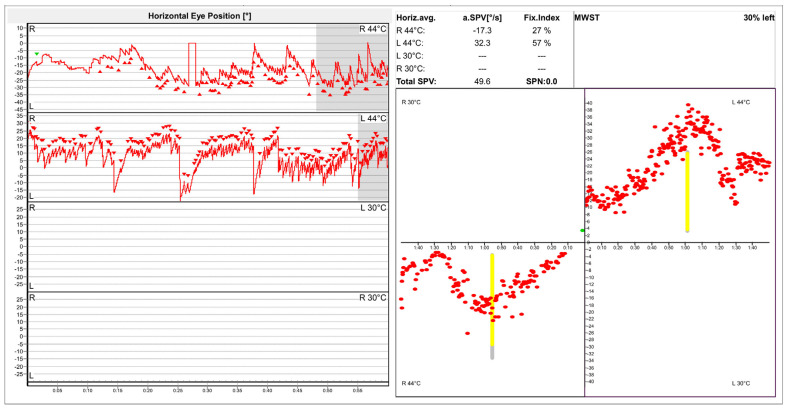
Monothermal caloric testing performed before intratympanic gentamicin therapy, showing preserved bilateral caloric reactivity. This baseline assessment was used as a screening evaluation of caloric responsiveness and should not be interpreted as a complete bithermal caloric test. The left panels show horizontal eye movement recordings during warm (44 °C) caloric irrigations. The right panels display the corresponding slow phase velocity (SPV) responses. Time is expressed in seconds and SPV in degrees per second (°/s). Caloric stimulation and eye movement recording were performed using an Interacoustics VNG system and caloric irrigator (Interacoustics A/S, Denmark).

**Figure 4:**
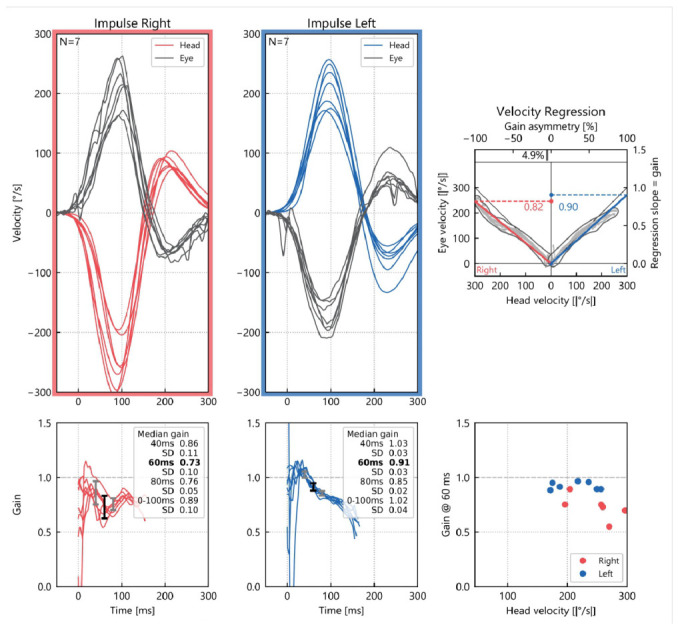
Video head impulse testing (vHIT) of the horizontal semicircular canals performed before intratympanic gentamicin therapy demonstrated preserved and relatively symmetrical vestibulo-ocular reflex (VOR) gain bilaterally, with a median VOR gain at 60 ms of 0.73 for the right horizontal canal and 0.91 for the left horizontal canal. No significant overt or covert corrective saccades were observed, consistent with normal lateral semicircular canal function. Testing was performed using an Interacoustics vHIT system (Interacoustics A/S, Denmark).

Before initiating gentamicin therapy, a comprehensive vestibular assessment was conducted. Videonystagmography (VNG) showed no spontaneous nystagmus. Caloric testing and the video head impulse test (vHIT) were within normal limits (Figure 2, Figure 3 and Figure 4). Pre-treatment monothermal caloric testing demonstrated preserved and approximately symmetrical bilateral caloric reactivity, without evidence of marked unilateral vestibular hypofunction. Because only monothermal stimulation was performed at baseline, this result was interpreted as a screening assessment and not as equivalent to a complete bithermal caloric evaluation. Video head impulse testing demonstrated preserved horizontal canal vestibulo-ocular reflex function bilaterally, with median gain values of approximately 0.82 for the right horizontal canal and 0.9 for the left horizontal canal, without significant corrective saccades, indicating no significant pre-treatment high-frequency horizontal canal deficit.

**Figure 5:**
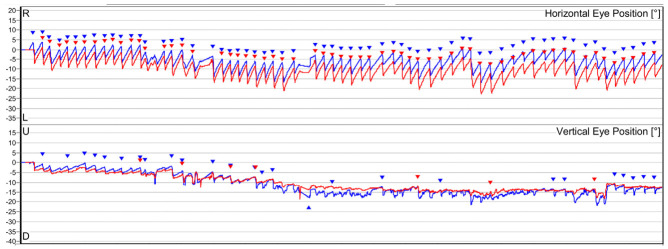
Video nystagmography (VNG) recording obtained after intratympanic gentamicin therapy, demonstrating intense spontaneous left-beating horizontal nystagmus, consistent with acute right-sided vestibular hypofunction induced by treatment. The upper panel shows horizontal eye position traces, while the lower panel shows vertical eye position recordings. Red and blue tracings represent recordings from each eye. The spontaneous nystagmus persisted in the absence of visual fixation and was associated with significant post-treatment vertigo and imbalance.

A low-dose titration strategy was used, consisting of sequential intratympanic gentamicin injections with clinical and vestibular reassessment before additional administration, aiming to achieve vertigo control while minimizing cochlear toxicity [7]. Gentamicin 40 mg/mL was injected transtympanically under microscopic guidance, with 0.5 mL administered at each session. The injections were administered at 7-day intervals. Before treatment, the patient was counseled regarding the expected vestibular effect of gentamicin, the possibility of prolonged imbalance during compensation, and the potential risk of cochlear toxicity and hearing deterioration. Treatment was stopped after the second injection, when the patient developed continuous vertigo and spontaneous left-beating nystagmus, consistent with the development of a right-sided vestibular deficit. In Figure 5, we present the VNG recording of the nystagmus.

**Figure 6:**
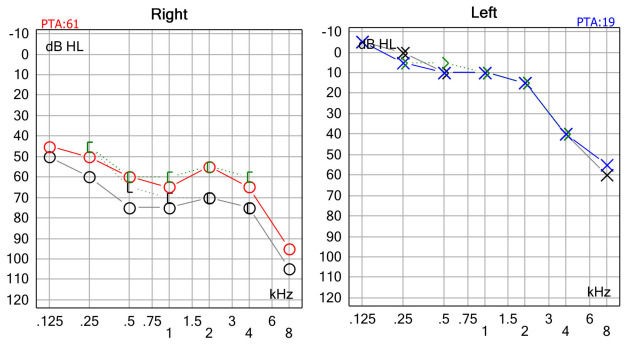
Pure-tone audiometry performed after the second intratympanic gentamicin injection. PTA for the left ear remained stable at 19 dB HL, whereas PTA for the right ear increased from 61 dB HL before treatment to 74 dB HL after treatment, indicating a worsening of hearing thresholds in the treated ear. Red circles and connecting lines represent baseline (pre-treatment) air-conduction thresholds of the right ear, while black circles and gray connecting lines represent post-treatment right-ear air-conduction thresholds. Blue crosses and connecting lines represent baseline (pre-treatment) air-conduction thresholds of the left ear, while black crosses and gray connecting lines represent post-treatment left-ear air-conduction thresholds. Green dashed lines and bracket symbols indicate bone-conduction thresholds. Frequencies are displayed in kHz on the horizontal axis and hearing thresholds in dB HL on the vertical axis.

The pure-tone audiometry we performed revealed a slight worsening of hearing thresholds compared with the pre-gentamicin baseline. The decline in hearing acuity predominantly affected high frequencies, as shown in Figure 6.

**Figure 7:**
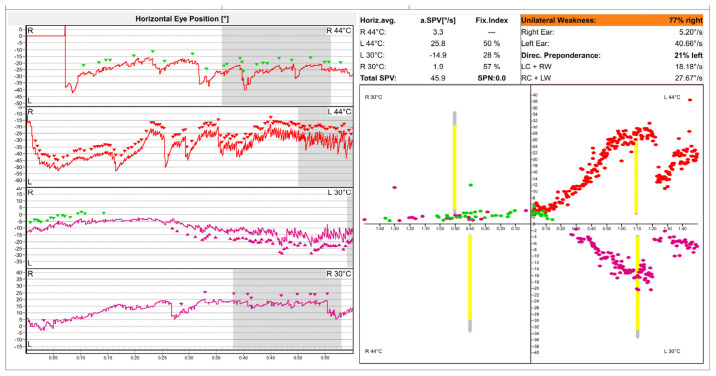
Bithermal caloric testing performed after intratympanic gentamicin therapy, demonstrating severe right-sided vestibular hypofunction, with markedly reduced caloric responses on the right side and preserved responses on the left. Residual spontaneous left-beating nystagmus was present during testing and contributed to baseline asymmetry. The left panels show horizontal eye position recordings during warm and cold irrigations, while the right panels summarize caloric response amplitudes and directional preponderance.

**Figure 8:**
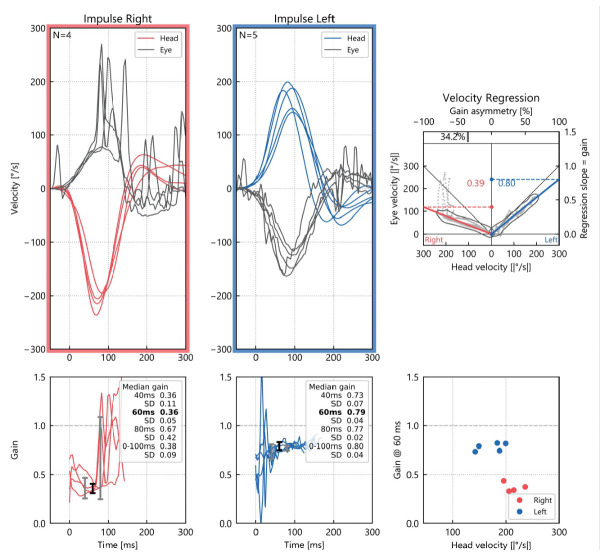
Video head impulse testing (vHIT) performed after intratympanic gentamicin therapy, demonstrating reduced vestibulo-ocular reflex (VOR) gain of the right lateral semicircular canal (median gain approximately 0.36–0.42) compared with preserved left-sided function (median gain approximately 0.73–0.80). Multiple overt corrective saccades were observed during rightward impulses, consistent with right unilateral vestibular hypofunction.

One month later, the patient still complained of dizziness but had no Tumarkin crises. The typical vertigo attacks of MD had completely resolved, with the patient reporting only residual dizziness. Nystagmus was still present, but only during examination with Frenzel goggles. We repeated the caloric testing, which demonstrated caloric areflexia in the right ear due to the effects of gentamicin (see Figure 7). After intratympanic gentamicin treatment, bedside head impulse testing showed visible corrective (catch-up) saccades during rightward head impulses. Video head impulse testing showed a reduced VOR gain on the right side, with both overt and covert corrective saccades during rightward head impulses, consistent with a unilateral vestibular deficit (see Figure 8).

### 2.5. Long-Term Follow-Up

**Figure 9:**
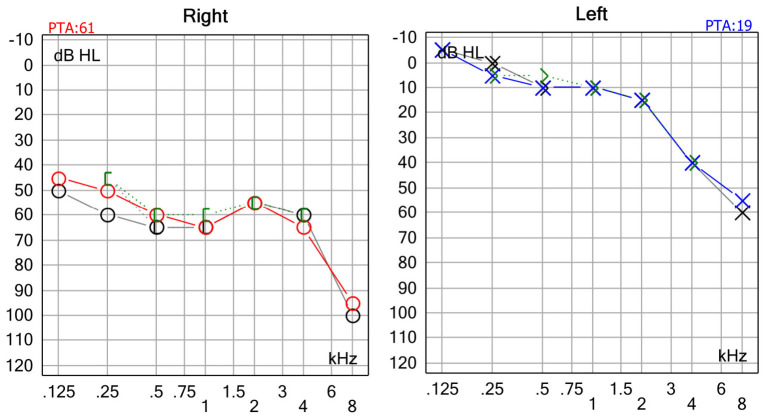
Pure-tone audiometry performed one year after intratympanic gentamicin therapy, demonstrating hearing thresholds comparable to those recorded before treatment. PTA for the right ear was 61 dB HL, and PTA for the left ear was 19 dB HL at follow-up. Red circles and connecting lines represent the baseline (pre-treatment) air-conduction thresholds of the right ear, while black circles and gray connecting lines represent the right-ear air-conduction thresholds recorded at the 1-year follow-up. Blue crosses and connecting lines represent baseline (pre-treatment) air-conduction thresholds of the left ear, while black crosses and gray connecting lines represent the left-ear air-conduction thresholds recorded at follow-up. Green dashed lines and bracket symbols indicate bone-conduction thresholds. Frequencies are displayed in kHz on the horizontal axis and hearing thresholds in dB HL on the vertical axis.

At the one-year follow-up, the patient experienced complete resolution of drop attacks and vertigo. She has been followed for two years with no recurrence of these symptoms. She remains on maintenance betahistine therapy. Interestingly, hearing thresholds returned close to baseline values during follow-up. However, this finding should be interpreted cautiously, as spontaneous hearing fluctuation related to Ménière’s disease and audiometric test–retest variability may have contributed to the apparent recovery (see Figure 9).

**Figure 10:**
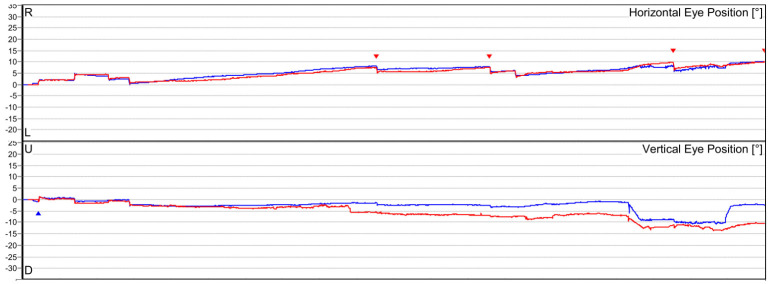
Video nystagmography (VNG) recording obtained one year after intratympanic gentamicin therapy, demonstrating a small residual spontaneous left-beating horizontal nystagmus, consistent with persistent but compensated right-sided vestibular hypofunction. Compared with the early post-treatment recordings, the amplitude and velocity of the spontaneous nystagmus were markedly reduced, suggesting partial central vestibular compensation over time. The upper panel shows horizontal eye position recordings, while the lower panel shows vertical eye position traces.

**Figure 11:**
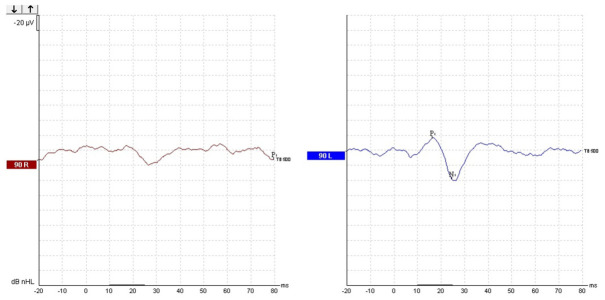
Cervical vestibular-evoked myogenic potential (cVEMP) testing performed one year after intratympanic gentamicin therapy. No reproducible P13–N23 waveform was identified in the right ear, consistent with right-sided saccular dysfunction. In contrast, the left ear demonstrated preserved biphasic P13–N23 responses with normal morphology and latency values.

The VNG performed at the one-year follow-up visit showed a small leftward nystagmus (see Figure 10), indicating that even after such a long period, the patient had not fully compensated for the right-sided deficit. Following the development of unilateral vestibular hypofunction, the patient underwent vestibular rehabilitation therapy aimed at promoting central vestibular compensation. The rehabilitation program included gaze stabilization exercises, vestibulo-ocular reflex adaptation exercises, postural control training, and balance exercises. Sessions were performed over several months, with home exercises encouraged between supervised rehabilitation visits. The main therapeutic goals were to reduce oscillopsia, improve gait stability, and facilitate functional recovery after gentamicin-induced vestibular hypofunction. Otherwise, she did not experience vertigo, only mild dizziness, and was very satisfied with the treatment results, mainly the disappearance of the vestibular drop attacks. To assess the slight instability the patient still reported, we also performed cervical vestibular-evoked myogenic potentials (cVEMPs) testing, as shown in Figure 11. Follow-up cVEMP testing demonstrated absent right-sided responses. However, because baseline otolithic testing was not available, it cannot be determined with certainty whether this finding was entirely treatment-related or partially associated with the underlying Ménière’s disease. A limitation of the present case is the absence of baseline cVEMP/oVEMP testing before intratympanic gentamicin administration. Therefore, the absent cVEMP response observed during follow-up cannot be conclusively attributed to gentamicin-induced otolithic dysfunction, since Ménière’s disease itself may involve the saccule and other otolithic structures. Given that the lack of response in the right ear persisted one year after the injection, this might explain the instability. Abnormal or absent cVEMP responses may contribute to persistent imbalance, as otolithic dysfunction has been associated with impaired postural stability and altered vestibulospinal control in Ménière’s disease {4}.

To improve the clarity of the clinical course, a chronological summary of symptom progression, treatments, serial audiovestibular assessments, vestibular rehabilitation, and follow-up findings was created. This overview highlights the relationship between therapeutic interventions and the evolution of clinical and vestibular findings throughout the disease course. The detailed timeline is presented in Table 1.

## 3. Discussion

A focused narrative review of the literature was conducted to contextualize current treatment strategies for vestibular drop attacks in Ménière’s disease and the role of audiovestibular monitoring after intratympanic gentamicin therapy. A structured search was performed in the PubMed and Scopus databases in January 2026 using combinations of the terms “Ménière’s disease”, “vestibular drop attacks”, “Tumarkin crisis”, “intratympanic gentamicin”, “video head impulse test”, “caloric testing”, and “vestibular-evoked myogenic potentials”. English-language articles reporting clinical cases, case series, original studies, or reviews relevant to vestibular drop attacks and vestibular assessment after gentamicin treatment were considered. Reference lists of selected articles were additionally screened to identify further relevant publications. Because of the limited literature and the narrative purpose of the review, a formal systematic review methodology was not applied.

VDAs, or Tumarkin crises, are rare but can be disabling symptoms of MD. Their reported occurrence varies widely, partly due to differing diagnostic criteria across studies, which range from postural disturbances and near-falls to sudden falls without warning or loss of consciousness. This difference between the diagnostic criteria for VDAs and the difficulty of observing the typical crisis in real life makes the true epidemiology of the phenomenon difficult to assess. A recent meta-analysis indicated a prevalence of 3% to 19% among MD patients [8], while Morales et al. observed that 13 out of 40 patients, monitored for up to 12 years, experienced drop attacks [9].

The mechanism behind Tumarkin crises is not fully understood. The most common hypothesis suggests that sudden abnormal stimulation or mechanical distortion of the otolithic organs leads to inappropriate activation of vestibulospinal pathways, causing an abrupt loss of postural tone while consciousness remains intact [10]. Some researchers have highlighted unstable otolithic function as a possible underlying cause [11]. These theories support the idea that VDAs result from sudden vestibular imbalance rather than true syncope or epileptic episodes.

The available literature on VDA treatment is limited and varied, as summarized in Table 2. Some research indicates that conservative approaches might be adequate for certain patients. Morales et al. noted that patients experiencing drop attacks did not need specialized treatment [9], and Janzen and Russell reported successful management of Tumarkin otolithic crises through conservative methods [12]. Conversely, other studies document frequent, traumatic, and disabling attacks that necessitate a more aggressive therapeutic approach.

In difficult-to-treat cases, intratympanic gentamicin is among the most reliably reported effective treatments. Murofushi et al. found it generally safe and effective for patients with intractable Menière’s disease or drop attacks [13]. Carmona et al. described success in managing refractory VDAs after betahistine therapy failed [14], while Wu et al. and Viana et al. also verified the long-term efficacy of gentamicin for drop attacks [15,16]. Additionally, case reports, including one by Dallan et al., support its use in severe cases [17]. Alternative approaches include controlled drug delivery through round-window microcatheters [18,19], posterior semicircular canal plugging [20], and vestibular neurotomy in carefully selected disabling cases [22].

This review reveals that objective audiovestibular monitoring has not been consistently reported in previous research. Most studies evaluated treatment success primarily by clinical improvement or symptom resolution, but detailed vestibular testing was often performed inconsistently. Some reports did not mention vestibular testing at all, while others included only specific tests such as audiometry, VEMPs, caloric testing, or MRI. This underscores the importance of the present case, where treatment response was tracked through serial clinical assessments, videonystagmography, caloric testing, vHIT, and pure-tone audiometry.

Interpreting vestibular testing in Menière’s disease (MD) requires caution. According to the 2020 AAO-HNS guidelines [5], routine vestibular tests or electrocochleography are not recommended solely for diagnosing MD, which remains mainly a clinical and audiometric diagnosis. Nonetheless, vestibular testing can be valuable when it impacts management decisions, especially before and after ablative treatments. In this case, audiovestibular monitoring directly supported treatment assessment by confirming both the effectiveness of gentamicin-induced vestibular hypofunction and the ongoing preservation of cochlear function.

Although the evidence supporting intratympanic gentamicin in Ménière’s disease remains limited and heterogeneous, this treatment continues to be widely used in clinical practice for patients with disabling, refractory vertigo or vestibular drop attacks when conservative measures and less destructive therapies have failed. In such severe cases, the potential benefit of vertigo control may outweigh the risk of cochlear toxicity, particularly when low-dose titration protocols and close audiovestibular monitoring are applied [24].

Gentamicin mainly affects the vestibular system, but cochlear toxicity is still a significant concern [25]. The degree of inner ear damage depends on factors such as dosage, the timing between injections, and potential drug buildup in the inner ear fluids. Consequently, treatment should target enough vestibular hypofunction to manage symptoms without causing excessive cochlear harm. Regular audiometry testing is crucial, especially since hearing loss may be temporary or worsen over time.

Vestibular testing can help quantify the extent of vestibular impairment caused by gentamicin. The presence of spontaneous nystagmus, head-shaking nystagmus, or a positive head impulse test is often used as an indicator of treatment effectiveness. Caloric testing can reveal diminished or absent vestibular responses post-treatment, while the vHIT offers additional insights into high-frequency vestibulo-ocular reflex function. VEMPs may also indicate otolithic involvement, although their use has varied across studies. Recent studies indicate that VEMPs are a useful method for assessing the response to gentamicin therapy [24,26]. The absence of VEMP potentials in the right ear in our case explains the difficult and prolonged compensation.

In this case, intratympanic gentamicin effectively resolved VDAs with lasting results. This was confirmed by objective measures, including intense spontaneous nystagmus beating to the left, a complete absence of the right-sided caloric response, and decreased right VOR gain on vHIT, along with overt and covert corrective saccades. Audiometric testing showed a temporary increase in hearing thresholds, mainly at high frequencies, which later returned to near baseline after one year. These results highlight the value of multimodal audiovestibular monitoring to verify the effectiveness of vestibular treatment while ensuring cochlear safety.

Overall, this case advocates for a step-by-step and personalized strategy for VDAs in MD. Conservative methods may be suitable at first, but persistent, traumatic, or disabling episodes might necessitate escalation to ablative or surgical options. Proper patient selection, early detection of VDAs, and vigilant audiovestibular monitoring are crucial to maximize treatment success and reduce associated risks.

One clinically relevant aspect highlighted by this case is the potential role of multimodal audiovestibular monitoring in increasing confidence in intratympanic gentamicin therapy. Although the procedure is effective in controlling vestibular drop attacks, clinicians may hesitate to recommend it because of concerns about hearing deterioration and the unpredictability of post-treatment vestibular changes. Serial vestibular and audiological assessments may provide a clearer understanding of treatment-related evolution and support individualized patient counseling and follow-up.

## 4. Conclusions

Vestibular drop attacks, although rare, are a serious complication of Ménière’s disease that can lead to disability and often require more aggressive treatment when conservative methods are ineffective. In stubborn cases, intratympanic gentamicin offers a reliable, long-lasting solution by creating a controlled unilateral vestibular deficit, effectively managing Tumarkin crises. This case underscores the significance of using multimodal audiovestibular monitoring during chemical vestibular ablation. Serial assessments—such as videonystagmography, caloric testing, and video head impulse testing—provided objective evidence of the gentamicin-induced vestibular deficit and verified the treatment’s success. Simultaneously, repeated pure-tone audiometry was crucial for ensuring cochlear safety, revealing only temporary hearing-threshold deterioration, with subsequent recovery to near-baseline levels over time.

This case highlights the importance of including objective vestibular and audiological assessments in treatment planning and ongoing monitoring for patients receiving intratympanic gentamicin. To our knowledge, few published studies have thoroughly documented longitudinal changes in vestibular function through multimodal audiovestibular testing following intratympanic gentamicin therapy for Tumarkin crises. Close monitoring can help improve treatment outcomes and reduce the risk of lasting hearing loss.

Comprehensive audiovestibular follow-up may help clinicians more accurately characterize treatment response after intratympanic gentamicin and reduce uncertainty about post-therapeutic functional outcomes.

## Figures and Tables

**Table 1:** Chronological summary of symptom progression, treatments, audiovestibular assessments, and follow-up findings.

Time	Clinical Events
Initial presentation	Definite unilateral Menière’s disease with recurrent vertigo, tinnitus, fluctuating hearing loss
Conservative treatment (6 months)	Betahistine, dietary measures
Intratympanic dexamethasone	Temporary symptom improvement
Recurrence (1 year)	Recurrent vestibular drop attacks (Tumarkin crisis)
Baseline testing	Audiometry, VNG, monothermal caloric testing, vHIT
Gentamicin injection 1	Low-dose intratympanic gentamicin
Gentamicin injection 2	Administered after persistent symptoms
Early post-treatment period (1 month)	Continuous vertigo, spontaneous nystagmus, imbalance
Follow-up vestibular testing	Caloric weakness, abnormal vHIT, absent cVEMP
Vestibular rehabilitation	Gaze stabilization and balance training
One-year follow-up post gentamicin	Complete control of vestibular drop attacks, hearing near baseline

**Table 2:** Summary of published studies on Tumarkin crises in Ménière’s disease, highlighting therapeutic strategies and the limited use of objective audiovestibular monitoring.

	Study	Study Design/Sample	Treatment Approach	Outcome on VDAs	Audiological Monitoring	Vestibular Monitoring	Imaging
1	Morales et al., 2005 [9]	Long-term observational study; 40 MD patients followed for 12 years	Conservative management	32.5% developed VDAs; none required specific intervention	Not reported	Clinical evaluation only	Not reported
2	Janzen et al. 1988 [12]	Observational study	Conservative management	Successful noninvasive management of VDAs	Not reported	Primarily clinical evaluation; no objective vestibular testing described	Not reported
3	Murofushi et al. 1997 [13]	Case series; 18 patients	Intratympanic gentamicin	Gentamicin considered effective and generally safe	Audiometry performed	Clinical vestibular assessment; no detailed vestibular instrumental monitoring	Not reported
4	Carmona et al. 2023 [14]	Retrospective cross-sectional study; 33 patients	Betahistine and intratympanic gentamicin	Gentamicin-controlled refractory VDAs	Not specifically reported	Demographic and symptom-based only; no vestibular testing performed	Not reported
5	Wu et al. 2019 [15]	Observational cohort; 177 MD patients; 16 with DA	Intratympanic gentamicin	Effective control of VDAs	Audiometry performed	VEMP testing, limited vestibular functional monitoring after treatment	MRI findings reported
6	Viana et al. 2014 [16]	Retrospective study; 24 ears	Intratympanic gentamicin	Long-term control of DAs	Limited audiological follow-up	Outcome mainly assessed clinically; limited objective vestibular testing reported	Not reported
7	Dallan et al. 2005 [17]	Case report	High-dose intratympanic gentamicin	Resolution of VDAs and vertigo	Audiometry performed	Caloric and ice-water testing performed	Not reported
8	Charabi et al. [18]/Thomsen et al. [19]	Technical reports	Gentamicin delivery via round-window microcatheter	Improved local drug delivery	Not emphasized	Focus on drug delivery rather than vestibular monitoring	Not reported
9	Zhang et al. [20]/Comacchio et al. [21]	Clinical studies	Posterior semicircular canal plugging	Reduction or resolution of VDAs	Hearing preservation discussed	Surgical outcomes emphasized; limited detailed vestibular monitoring	Not reported
10	Gentine et al. 2008 [22]	Clinical series	Lateral semicircular canal plugging	Effective for incapacitating MD, less suitable for VDAs	Hearing outcomes reported	Clinical outcome assessment predominated	Not reported
11	Velaine et al. 2022 [23]	Retrospective study	Vestibular neurotomy	Long -term control of vertigo and VDAs	Hearing preservation assessed	Clinical follow-up with hearing preservation assessment	Not reported

## Data Availability

The data are not publicly available due to patient privacy and ethical restrictions. The original contributions presented in this study are included in the article. Further inquiries can be directed to the corresponding author.

## Abbreviations

The following abbreviations are used in this manuscript:

AAO-HNS	American Academy of Otolaryngology–Head and Neck Surgery
DAs	Drop attacks
ENT	Ear, nose, and throat
IT	Intratympanic
MD	Ménière’s disease
PTA	Pure-tone average
SNHL	Sensorineural hearing loss
VDAs	Vestibular drop attacks
VEMP	Vestibular-evoked myogenic potentials
cVEMPs	Cervical vestibular-evoked myogenic potentials
VNG	Videonystagmography
vHIT	Video head impulse test
VOR	Vestibulo-ocular reflex
